# Development and Validation of an Integrated Suite of Prediction Models for All-Cause 30-Day Readmissions of Children and Adolescents Aged 0 to 18 Years

**DOI:** 10.1001/jamanetworkopen.2022.41513

**Published:** 2022-11-11

**Authors:** Denise M. Goodman, Mia T. Casale, Karen Rychlik, Michael S. Carroll, Katherine A. Auger, Tracie L. Smith, Jenifer Cartland, Matthew M. Davis

**Affiliations:** 1 Division of Critical Care Medicine, Ann & Robert H. Lurie Children’s Hospital of Chicago, Chicago, Illinois; 2 Department of Pediatrics, Northwestern University Feinberg School of Medicine, Chicago, Illinois; 3 Data Analytics and Reporting, Ann & Robert H. Lurie Children’s Hospital of Chicago, Chicago, Illinois; 4 Biostatistics Research Core, Stanley Manne Children’s Research Institute, Ann & Robert H. Lurie Children’s Hospital of Chicago, Chicago, Illinois; 5 Currently serving as an independent consultant; 6 Division of Hospital Medicine, Cincinnati Children’s Hospital Medical Center, Cincinnati, Ohio; 7 Department of Pediatrics, University of Cincinnati College of Medicine, Cincinnati, Ohio; 8 Mary Ann & J. Milburn Smith Child Health Outcomes, Research, and Evaluation Center, Stanley Manne Children’s Research Institute, Ann & Robert H. Lurie Children’s Hospital of Chicago, Chicago, Illinois; 9 Currently retired; 10 Division of Advanced General Pediatrics and Primary Care, Ann & Robert H. Lurie Children’s Hospital of Chicago, Chicago, Illinois; 11 Department of Medicine, Northwestern University Feinberg School of Medicine, Chicago, Illinois; 12 Department of Medical Social Sciences, Northwestern University Feinberg School of Medicine, Chicago, Illinois; 13 Department of Preventive Medicine, Northwestern University Feinberg School of Medicine, Chicago, Illinois

## Abstract

**Importance:**

Readmission is often considered a hospital quality measure, yet no validated risk prediction models exist for children.

**Objective:**

To develop and validate a tool identifying patients before hospital discharge who are at risk for subsequent readmission, applicable to all ages.

**Design, Setting, and Participants:**

This population-based prognostic analysis used electronic health record–derived data from a freestanding children’s hospital from January 1, 2016, to December 31, 2019. All-cause 30-day readmission was modeled using 3 years of discharge data. Data were analyzed from June 1 to November 30, 2021.

**Main Outcomes and Measures:**

Three models were derived as a complementary suite to include (1) children 6 months or older with 1 or more prior hospitalizations within the last 6 months (recent admission model [RAM]), (2) children 6 months or older with no prior hospitalizations in the last 6 months (new admission model [NAM]), and (3) children younger than 6 months (young infant model [YIM]). Generalized mixed linear models were used for all analyses. Models were validated using an additional year of discharges.

**Results:**

The derivation set contained 29 988 patients with 48 019 hospitalizations; 50.1% of these admissions were for children younger than 5 years and 54.7% were boys. In the derivation set, 4878 of 13 490 admissions (36.2%) in the RAM cohort, 2044 of 27 531 (7.4%) in the NAM cohort, and 855 of 6998 (12.2%) in the YIM cohort were followed within 30 days by a readmission. In the RAM cohort, prior utilization, current or prior procedures indicative of severity of illness (transfusion, ventilation, or central venous catheter), commercial insurance, and prolonged length of stay (LOS) were associated with readmission. In the NAM cohort, procedures, prolonged LOS, and emergency department visit in the past 6 months were associated with readmission. In the YIM cohort, LOS, prior visits, and critical procedures were associated with readmission. The area under the receiver operating characteristics curve was 83.1 (95% CI, 82.4-83.8) for the RAM cohort, 76.1 (95% CI, 75.0-77.2) for the NAM cohort, and 80.3 (95% CI, 78.8-81.9) for the YIM cohort.

**Conclusions and Relevance:**

In this prognostic study, the suite of 3 prediction models had acceptable to excellent discrimination for children. These models may allow future improvements in tailored discharge preparedness to prevent high-risk readmissions.

## Key Points

**Question:**

Can a readmission risk assessment score be developed to apply to all children that could be implemented in real time in the electronic health record (EHR)?

**Findings:**

In this prognostic study that included 48 019 admissions, a suite of 3 age- and utilization-specific models was developed and validated with ongoing length of stay, use of specific therapies, and past utilization as predictive factors for all-cause 30-day readmission. Performance ranged from acceptable to excellent.

**Meaning:**

These findings suggest that validated models can subsequently be implemented into the EHR using readily available EHR data to reliably identify readmission risk.

## Teaser

This prognostic study develops pediatric readmission risk tools that use clinical, demographic, and socioeconomic data readily available in the electronic health record and reflect the dynamic trajectory of illness and clinical care throughout the hospital stay among children and adolescents.

## Introduction

Hospital readmission rate is widely viewed as a measure of quality of care.^1,2^ In the pediatric literature, low hospital volumes for children,^3^ readmission to the same vs different hospitals,^4^ and questions about clinically plausible preventability^5,6^ have prompted criticism of this metric as a quality indicator. If hospitals were able to predict readmission risk during inpatient stays, they could more effectively target in-hospital case management and plan postdischarge follow-up.^7^ Accurate readmission prediction could thereby shift the focus from assessment of readmission preventability toward a more proactive stance of discharge readiness.

Published risk scores for adults predict hospital readmissions,^8,9^ 30-day mortality on admission,^10,11^ or both^12,13^ but have limited utility for pediatric patients. Many of these tools also reflect cross-sectional patient and hospital factors (eg, at hospital admission^11^), rather than longitudinal data updated during a hospital stay. In addition, some models use variables that are only available retrospectively, such as ultimate length of stay (LOS) of the index admission. The need for a pediatric readmission risk model is well-recognized,^7,14^ and one such model was described in 2018.^15^ However, that model relied on a proprietary measure of clinical status (the pediatric Rothman index) that requires a paid subscription. Another published screening tool had low discrimination performance.^16^ No risk score is available for young infants, who may have different risk factors for readmission. Finally, no other models create separate cohorts based on recent hospitalization.

We sought to develop pediatric readmission risk tools that would (1) use clinical, demographic, and socioeconomic data readily available in the electronic health record (EHR) without manual extraction of data or integration of external data sets and (2) reflect the dynamic trajectory of illness and clinical care throughout the hospital stay. Further, we elected to use variables in the EHR that would be available in real time such that the risk prediction models could be used during a hospital admission to inform discharge planning before the patient leaves the hospital.^7,17^

## Methods

### Study Design

We conducted a prognostic study to derive readmission risk prediction models from 3 years of discharges (January 1, 2016, to December 31, 2018) at Ann & Robert H. Lurie Children’s Hospital of Chicago (LCH). Each hospital encounter (observation or inpatient) was considered an index admission. The primary outcome was all-cause 30-day readmission. As such, some encounters served both as a single (index) admission and as a readmission if an antecedent admission occurred less than 30 days previously. We subsequently validated the derived models in an additional year of data (January 1 to December 31, 2019). This study was reviewed by the institutional review board of LCH and deemed exempt from review and the requirement for informed consent because it used EHRs. This study adhered to Transparent Reporting of a Multivariable Prediction Model for Individual Prognosis or Diagnosis (TRIPOD) reporting guideline.^18^

### Setting

Ann & Robert H. Lurie Children’s Hospital is a 364-bed independent children’s hospital in a large, diverse metropolitan area. There are 3 intensive care units (cardiac, pediatric, and neonatal, with full complements of pediatric, surgical, anesthesia, and medical imaging specialists immediately available). Psychiatric admissions are excluded because most occur in a separate psychiatric unit. However, medical admissions for psychiatric conditions (ie, ingestions) were included.

### Model Derivation

For derivation cohorts, we excluded hospitalizations during which patients died or were transferred to another hospital (n = 652), because readmission patterns would be potentially different for these children. Likewise, we excluded patients with an address outside the US or if all geographic information was missing (n = 185), because we assumed these patients would have different readmission patterns. We did not exclude patients with partially missing geographic elements if their zip code was in the US. Exclusions totaled 835 (1.3% of admissions). eMethods 1 in the Supplement details how deaths (376 admissions [0.6%]) were handled; eMethods 2 in the Supplement details how missing data were handled.

We purposively sought to develop 3 different models as a complementary suite to estimate readmission risk for distinct patient groups. Recognizing that children with prior utilization have both a higher readmission risk and may have different transition needs, we separated children with prior hospitalization in the preceding 6 months from those without. The recent admission model (RAM) was designed to calculate readmission risk in patients who had been hospitalized in the preceding 6 months. The new admission model (NAM) was designed to assess readmission risk in children without a recent hospitalization. The young infant model (YIM) was designed to assess risk for children too young (<6 months) to have a comparable history of utilization.

### Candidate Variables

A list of candidate variables was developed through review of prior published readmission models^8,9,10,11,12,13,14,15^ and clinical judgment of the authors. Individual demographic variables included sex, age, race and ethnicity, preferred language, insurance, and distance to LCH. Race and ethnicity data were obtained from the EHR. Although our policy is for caregivers to self-identify race and ethnicity at registration, we cannot guarantee this is universally done. Race and ethnicity were included as a single combined variable (Hispanic ethnicity, non-Hispanic Black and non-Hispanic White race, non-Hispanic other races and ethnicities [including American Indian or Alaska Native, Asian, Native Hawaiian or other Pacific Islander, other race or ethnicity, and declined to answer], and unknown), because children who are members of ethnic or racial minority groups likely experience disparate access to outpatient care due to structural racism and implicit bias that may be associated with readmission risk. Data on sex were self-reported by caregivers.

For measures of socioeconomic status (SES), we used median values for the patient’s residential zip code for educational level, employment, and income from 2011-2015 American Community Survey 5-year estimates^19^ to dichotomize each into high and low categories. For medical history and complexity, we assessed prior acute hospital-level utilization (hospital and emergency department discharges) at LCH within 6 months preceding the index visit. As a proxy for medical complexity, we considered exposure to at least 1 recent procedure (invasive or noninvasive ventilation, central venous catheter placement, or transfusion) within the last 6 months or during the current stay. These procedures were widely identified in a cohort of patients hospitalized with complex chronic illness^20^ and could be extracted in real time, as opposed to *International Statistical Classification of Diseases and Related Health Problems, Tenth Revision–*based taxonomies.^21,22,23^ Finally, we assessed whether the patient had a primary care physician listed in the EHR.

Features of the index admission included principal diagnosis, LOS, admission source (emergency department vs other admission type), surgical vs medical (based on admitting service), care in the pediatric intensive care unit during the current admission, the neonatal intensive care unit, the cardiac intensive care unit, and calendar season. We categorized principal diagnosis into 10 common categories and one “other” category for each model cohort (eTable 1 in the Supplement). We identified the most frequent diagnosis categories in both the readmitted patients and the cohort as a whole, creating a final consolidated list (details in eMethods 3 in the Supplement). For all inpatient encounters, we used primary *International Classification of Diseases*–coded diagnosis. For LOS, because it is only known at the end of a stay and this prediction model can be applied in real time during hospitalization, we used a dichotomous indicator for prolonged LOS defined as exceeding the median LOS in the derivation cohort (≥4 days for RAM and ≥3 days for NAM and YIM).

### Statistical Analysis

Data were analyzed from June 1 to November 30, 2021. We used sequential time epochs for the LCH derivation (2016-2018) and validation (2019) sets. TRIPOD recommends that data sets be split temporally rather than randomly.^23^ For the derivation of each of the 3 models, we fit a series of generalized mixed linear models (GMLM) for bivariate comparisons to minimize the use of variables that have little or no impact on the model, using *P* < .10 and an odds ratio of at least 1.10 or no greater than 0.90 in at least one of the categories in each predictor variable. A variance components covariance structure was used to build the models. All covariate categories were compared with a reference group, consistently chosen as the largest category within the variable. The GMLM also included a random participant effect to account for within-patient correlations of measurements for patients who were admitted more than once; random effects were selected to account for changes in age-related epidemiology of illness for patients with more than 1 admission. The 3 models were fitted separately. Our final multivariable models from the derivation cohort retained covariates with 2-sided *P* < .05 in each of the 3 GMLM models. The predicted probabilities for readmission in each of the 3 models were used to estimate the odds of all-cause 30-day readmission. The area under the receiver operating characteristics curve (AUROC) (equivalent to *C* statistic)^24^ and area under the precision-recall curve (which reduces the risk of overestimating performance with unbalanced data sets) were calculated for each model.

For the validation models, we performed GMLM with the selected covariates from each of the 3 final models (RAM, NAM, and YIM) from the derivation cohort. Summary *C* statistics were then recalculated on these validation cohorts. All analyses were performed using SAS, version 9.4 (SAS Institute Inc).

## Results

The derivation set included 29 988 patients who contributed 48 019 hospital discharges; 50.1% of discharges were of children younger than 5 years; 54.7% were boys and 45.3% were girls; 54.1% were covered by Medicaid; and 59.8% had no admission in the preceding 30 days (eTable 2 in the Supplement). The validation set included 12 617 patients with 17 486 hospital discharges; the discharges in the validation set had similar characteristics, with 50.6% of discharges of children younger than 5 years; 54.5% were boys and 45.5% were girls; and 53.9% were covered by Medicaid (eTable 2 in the Supplement). Regarding subgroups of patients (RAM, NAM, and YIM cohorts) as described in the Methods section, in the derivation set readmission proportions were 4878 of 13 490 (36.2%) for RAM, 2044 of 27 531 (7.4%) for NAM, and 855 of 6998 (12.2%) for YIM (Table 1). Table 1 presents other sociodemographic and clinical characteristics of the derivation cohort. In the validation set, readmission proportions were 1687 of 4843 (34.8%) for RAM, 793 of 10 161 (7.8%) for NAM, and 275 of 2482 (11.1%) for YIM.

**Table 1.** Patient Encounter Descriptive Statistics

Variable	Model cohort^a^		
	RAM (n = 13 490)	NAM (n = 27 531)	YIM (n = 6998)
Primary outcome			
No subsequent admission within 30 d	8612 (63.8)	25 487 (92.6)	6143 (87.8)
Subsequent admission within 30 d	4878 (36.2)	2044 (7.4)	855 (12.2)
Age at admission^b^			
<2 mo	NA	NA	3871 (55.3)
2-5 mo	NA	NA	3127 (44.7)
6-11 mo	1192 (8.8)	2282 (8.3)	NA
1-4 y	4394 (32.6)	9195 (33.4)	NA
5-14 y	4877 (36.2)	11 483 (41.7)	NA
≥15 y	3027 (22.4)	4571 (16.6)	NA
Cardiac critical care unit admission			
No	12 937 (95.9)	26 457 (96.1)	6339 (90.6)
Yes	553 (4.1)	1074 (3.9)	659 (9.4)
ED admittance			
No	6471 (48.0)	11 840 (43.0)	3387 (48.4)
Yes	7019 (52.0)	15 691 (57.0)	3611 (51.6)
Insurance			
Government^c^	7381 (54.7)	14 946 (54.3)	3648 (52.1)
Commercial^d^	5990 (44.4)	12 302 (44.7)	3314 (47.4)
Other or unspecified	119 (0.9)	283 (1.0)	36 (0.5)
Length of stay, d			
0-2	NA	17 738 (64.4)	3528 (50.4)
0-3	8027 (59.5)	NA	NA
≥3	NA	9793 (35.6)	3470 (49.6)
≥4	5463 (40.5)	NA	NA
PICU admission			
No	12 643 (93.7)	26 528 (96.4)	6875 (98.2)
Yes	847 (6.3)	1003 (3.6)	123 (1.8)
Primary care physician assigned			
No	748 (5.5)	3822 (13.9)	1728 (24.7)
Yes	12 742 (94.5)	23 709 (86.1)	5270 (75.3)
Primary diagnosis category			
ALTE/BRUE	NA	NA	135 (1.9)
Appendicitis	69 (0.5)	857 (3.1)	NA
Asthma	588 (4.4)	2376 (8.6)	NA
Cardiac	NA	NA	406 (5.8)
CNS shunt	183 (1.4)	161 (0.6)	NA
Congenital anomalies	NA	NA	472 (6.7)
Dehydration/GI tract infection	401 (3.0)	1123 (4.1)	NA
Esophageal reflux	NA	NA	132 (1.9)
Fever	187 (1.4)	122 (0.4)	278 (4.0)
Neonatal jaundice	NA	NA	240 (3.4)
Pyloric stenosis	NA	NA	151 (2.2)
Respiratory tract			
Lower			
Bronchiolitis or pneumonia	NA	NA	1326 (19.0)
Bronchiolitis	478 (3.5)	1362 (4.9)	NA
Pneumonia	846 (6.3)	1591 (5.8)	NA
Upper	235 (1.7)	638 (2.3)	254 (3.6)
Seizure	641 (4.8)	1508 (5.5)	NA
Sickle cell disease	333 (2.5)	231 (0.8)	NA
UTI and pyelonephritis	NA	NA	196 (2.8)
Other	9529 (70.6)	17 562 (63.8)	3408 (48.7)
Prior select procedures in the 6 mo before admission			
0	6839 (50.7)	25 748 (93.5)	5917 (84.6)
≥1 Select procedures	6651 (49.3)	1783 (6.5)	1081 (15.4)
Prior utilization in the 6 mo before admission^e^			
0	NA	23 386 (84.9)	5331 (76.2)
1	4505 (33.4)	NA	1081 (15.4)
≥1	NA	4145 (15.1)	NA
2	2862 (21.2)	NA	338 (4.8)
3	1806 (13.4)	NA	NA
≥3	NA	NA	248 (3.5)
≥4	4317 (32.0)	NA	NA
Race and ethnicity			
Hispanic, any race	4469 (33.1)	9803 (35.6)	2356 (33.7)
Non-Hispanic Black	2445 (18.1)	4730 (17.2)	1143 (16.3)
Non-Hispanic White	5374 (39.8)	10 258 (37.3)	2728 (39.0)
Non-Hispanic other^f^ or unknown/unspecified	1202 (8.9)	2740 (10.0)	771 (11.0)
Season of admission			
December, January, or February	3358 (24.9)	7138 (25.9)	2186 (31.2)
March, April, or May	3633 (26.9)	7107 (25.8)	1674 (23.9)
June, July, or August	3264 (24.2)	6341 (23.0)	1504 (21.5)
September, October, or November	3235 (24.0)	6945 (25.2)	1634 (23.3)
Educational level^g^			
<90% With high school degree or higher, or unknown	7434 (55.1)	16 003 (58.1)	4125 (58.9)
≥90% With high school degree or higher	6056 (44.9)	11 528 (41.9)	2873 (41.1)
Income^g^			
<$50 000 or unknown	3423 (25.4)	7549 (27.4)	1935 (27.7)
≥$50 000	10 067 (74.6)	19 982 (72.6)	5063 (72.3)
Service category			
Medical	11 358 (84.2)	20 105 (73.0)	5834 (83.4)
Surgical	2132 (15.8)	7426 (27.0)	1164 (16.6)

Abbreviations: ALTE/BRUE, apparent life-threatening event/brief resolved unexplained event; CNS, central nervous system; ED, emergency department; GI, gastrointestinal; NA, not applicable; NAM, new admission model; PICU, pediatric intensive care unit; RAM, recent admission model; UTI, urinary tract infection; YIM, young infant model.

^a^ Unless otherwise indicated, data are expressed as No. (%) of admissions.

^b^ Aggregated into clinically relevant categories in RAM and NAM cohorts. For the YIM cohort, the age cut point was established empirically based on age distribution during initial bivariate analysis.

^c^ Includes Medicare, Medicaid, accountability care entity, care coordination entity, CHAMPUS (Civilian Health and Medical Program of the Uniformed Services), or other government insurance.

^d^ Includes Blue Cross/Blue Shield, managed care, or other commercial insurance.

^e^ Defined as an inpatient stay, observation, or ED visit. In the NAM cohort, prior utilization pertains to ED visits only, because by definition this population includes only encounters for which there is no previous hospitalization.

^f^ Includes American Indian or Alaska Native, Asian, Native Hawaiian or other Pacific Islander, other race or ethnicity, and declined to answer.

^g^ Based on zip code.

eTables 3 to 5 in the Supplement present the bivariate (unadjusted) associations between predictor variables and outcomes for each of the 3 derivation models. Three variables were excluded from further consideration in multivariable analyses because no bivariate associations were found: sex, preferred language, and whether the patient was ever in the neonatal intensive care unit.

For the RAM subsample (Table 2), variables with the highest odds of readmission were increasing frequency of prior utilization (adjusted odds ratio [aOR] for ≥4 utilizations, 2.30 [95% CI, 2.06-2.58]), procedures (eg, transfusion, ventilation, or central venous catheter) (aOR, 2.35 [95% CI, 2.13-2.60]), and LOS exceeding the median (aOR, 1.17 [95% CI, 1.08-1.28]). Commercial insurance (aOR, 1.18 [95% CI, 1.07-1.30]) and admission from a site other than the emergency department (aOR, 1.13 [95% CI, 1.03-1.23]) were also associated with greater odds of readmission, whereas surgical admission was associated with lower odds (aOR, 0.55 [95% CI, 0.48-0.63]). Certain common diagnosis categories such as asthma (aOR, 0.35 [95% CI, 0.26-0.47]), lower respiratory tract conditions (eg, aOR for bronchiolitis, 0.64 [95% CI, 0.50-0.83]), seizure (aOR, 0.59 [95% CI, 0.47-0.74]), and sickle cell crisis (aOR, 0.69 [95% CI, 0.50-0.96]) were also associated with lower odds of readmission compared with the reference group.

**Table 2.** Multivariable Analysis of Recent Admission Model Cohort

Variable	Estimate (SE)	aOR (95% CI)	*P* value
Intercept	−1.68 (0.06)	NA	<.001
ED admittance			
No	0.12 (0.05)	1.13 (1.03-1.23)	.009
Yes	0	1 [Reference]	NA
Insurance			
Commercial^a^	0.16 (0.05)	1.18 (1.07-1.30)	.001
Other or unspecified	−0.58 (0.26)	0.56 (0.33-0.94)	.03
Government^b^	0	1 [Reference]	NA
Length of stay, d			
≥4	0.16 (0.04)	1.17 (1.08-1.28)	<.001
0-3	0	1 [Reference]	NA
Primary diagnosis category			
Appendicitis	−0.10 (0.37)	0.91 (0.44-1.89)	.80
Asthma	−1.06 (0.16)	0.35 (0.26-0.47)	<.001
CNS shunt	0.29 (0.20)	1.34 (0.90-1.99)	.15
Dehydration/GI tract infection	−0.19 (0.13)	0.82 (0.64-1.06)	.13
Fever	−0.30 (0.18)	0.74 (0.52-1.05)	.09
Respiratory tract			
Lower			
Bronchiolitis	−0.44 (0.13)	0.64 (0.50-0.83)	<.001
Pneumonia	−0.40 (0.09)	0.67 (0.56-0.80)	<.001
Upper	−0.12 (0.16)	0.89 (0.65-1.22)	.46
Seizure	−0.53 (0.12)	0.59 (0.47-0.74)	<.001
Sickle cell disease	−0.37 (0.17)	0.69 (0.50-0.96)	.03
Other	0	1 [Reference]	NA
Prior select procedure in the 6 mo before admission			
≥1	0.86 (0.05)	2.35 (2.13-2.60)	<.001
0	0	1 [Reference]	NA
Prior utilization in 6 mo before admission^c^			
≥4	0.83 (0.06)	2.30 (2.06-2.58)	<.001
3	0.52 (0.07)	1.69 (1.48-1.93)	<.001
2	0.28 (0.06)	1.32 (1.18-1.49)	<.001
1	0	1 [Reference]	NA
Service category			
Surgical	−0.60 (0.07)	0.55 (0.48-0.63)	<.001
Medical	0	1 [Reference]	NA

Abbreviations: aOR, adjusted odds ratio; CNS, central nervous system; GI, gastrointestinal; NA, not applicable.

^a^ Includes Blue Cross/Blue Shield, managed care, or other commercial insurance.

^b^ Includes Medicare, Medicaid, accountability care entity, care coordination entity, CHAMPUS (Civilian Health and Medical Program of the Uniformed Services), or other government insurance.

^c^ Defined as an inpatient stay, observation, or emergency department visit.

For the NAM model (Table 3), LOS (aOR, 2.26 [95% CI, 2.05-2.49]) and prior procedures (aOR, 2.10 [95% CI, 1.82-2.42]) were also associated with increased risk of readmission. In addition, prior emergency department utilization (aOR, 1.32 [95% CI, 1.16-1.50]) and diagnoses of central nervous system (CNS) shunt infection or malfunction (aOR, 2.15 [95% CI, 1.37-3.38]) and sickle cell crisis (aOR, 1.55 [95% CI, 1.04-2.32]) (compared with the reference group) were associated with higher odds of readmission. Children hospitalized with several diagnosis categories (appendicitis, asthma, dehydration, lower respiratory tract infections, and seizure) had lower odds of readmission. Likewise, readmissions were less likely for non-Hispanic Black (aOR, 0.78 [95% CI, 0.68-0.90]) or Hispanic (aOR, 0.82 [95% CI, 0.74-0.92]) children than for non-Hispanic White children.

**Table 3.** Multivariable Analysis of New Admission Model Cohort

Variable	Estimate (SE)	aOR (95% CI)	*P* value
Intercept	−2.70 (0.05)	NA	<.001
ED admittance			
No	−0.15 (0.05)	0.86 (0.78 to 0.95)	.003
Yes	0	1 [Reference]	NA
Length of stay, d			
≥3	0.82 (0.05)	2.26 (2.05 to 2.49)	<.001
0	0	1 [Reference]	NA
Primary diagnosis category			
Appendicitis	−0.62 (0.18)	0.54 (0.38 to 0.76)	<.001
Asthma	−0.69 (0.12)	0.50 (0.40 to 0.64)	<.001
CNS shunt	0.77 (0.23)	2.15 (1.37 to 3.38)	<.001
Dehydration/GI tract infection	−0.45 (0.15)	0.64 (0.48 to 0.85)	.003
Fever	0.09 (0.34)	1.10 (0.57 to 2.13)	.79
Respiratory tract			
Lower			
Bronchiolitis	−0.29 (0.12)	0.75 (0.59 to 0.94)	.01
Pneumonia	−0.33 (0.10)	0.72 (0.59 to 0.88)	.002
Upper	−0.29 (0.19)	0.75 (0.52 to 1.08)	.13
Seizure	−0.27 (0.12)	0.76 (0.60 to 0.97)	.03
Sickle cell disease	0.44 (0.20)	1.55 (1.04 to 2.32)	.03
Other	0	1 [Reference]	NA
Prior select procedure in the 6 mo before admission			
≥1	0.74 (0.07)	2.10 (1.82 to 2.42)	<.001
0	0	1 [Reference]	NA
Prior utilization in the 6 mo before admission^a^			
≥1	0.28 (0.06)	1.32 (1.16 to 1.50)	<.001
0	0	1 [Reference]	NA
Race and ethnicity			
Hispanic	−0.19 (0.06)	0.82 (0.74 to 0.92)	.001
Non-Hispanic Black	−0.24 (0.07)	0.78 (0.68 to 0.90)	.001
Non-Hispanic White	0	1 [Reference]	NA
Non-Hispanic other^b^ or unknown/unspecified	−0.25 (0.09)	0.78 (0.66 to 0.92)	.004

Abbreviations: aOR, adjusted odds ratio; CNS, central nervous system; ED, emergency department; GI, gastrointestinal; NA, not applicable.

^a^ Pertains to ED visits only, because by definition this population includes only encounters for which there is no previous hospitalization.

^b^ Includes American Indian or Alaska Native, Asian, Native Hawaiian or other Pacific Islander, other race or ethnicity, and declined to answer.

In the YIM model (Table 4), prolonged LOS (aOR, 1.69 [95% CI, 1.43-2.00]), prior health care utilization (aOR for ≥3, 3.08 [95% CI, 2.26-4.19]), and prior procedures (aOR, 1.43 [95% CI, 1.18-1.74]) were associated with higher readmission risk. There were no diagnoses associated with increased risk for readmission, although several diagnosis categories were associated with lower risk than the reference group.

**Table 4.** Multivariable Analysis of Young Infant Model Cohort

Variable	Estimate (SE)	aOR (95% CI)	*P* value
Intercept	−2.43 (0.08)	NA	<.001
Length of stay, d			
≥3	0.53 (0.08)	1.69 (1.43 to 2.00)	<.001
0-2	0	1 [Reference]	NA
Primary diagnosis category			
ALTE/BRUE	0.02 (0.31)	1.02 (0.56 to 1.85)	.96
Cardiac	0.06 (0.15)	1.06 (0.79 to 1.42)	.71
Congenital anomalies	−0.17 (0.15)	0.85 (0.63 to 1.15)	.28
Esophageal reflux	0.13 (0.27)	1.14 (0.68 to 1.92)	.61
Fever	−0.82 (0.29)	0.44 (0.25 to 0.78)	.004
Neonatal jaundice	−1.18 (0.39)	0.31 (0.14 to 0.66)	.003
Pyloric stenosis	−0.62 (0.35)	0.54 (0.27 to 1.08)	.08
Respiratory tract			
Upper	−0.56 (0.25)	0.57 (0.35 to 0.94)	.03
Lower (bronchiolitis and pneumonia)	−0.49 (0.11)	0.61 (0.49 to 0.76)	<.001
UTI and pyelonephritis	−0.68 (0.29)	0.51 (0.29 to 0.90)	.02
Other	0	1 [Reference]	NA
Prior procedure in the 6 mo before admission			
≥1	0.36 (0.10)	1.43 (1.18 to 1.74)	<.001
0	0	1 [Reference]	NA
Prior utilization in the 6 mo before admission^a^			
≥3	1.12 (0.16)	3.08 (2.26 to 4.19)	<.001
2	0.82 (0.14)	2.27 (1.72 to 3.01)	<.001
1	0.43 (0.10)	1.54 (1.27 to 1.88)	<.001
0	0	1 [Reference]	NA

Abbreviations: ALTE/BRUE, apparent life-threatening event/brief resolved unexplained event; aOR, adjusted odds ratio; NA, not applicable; UTI, urinary tract infection.

^a^ Defined as an inpatient stay, observation, or emergency department visit.

Model discrimination across the suite of 3 models (Figure and eTable 6 in the Supplement) ranged from acceptable to excellent. The RAM model with AUROC of 83.1 (95% CI, 82.4-83.8) and YIM model with AUROC of 80.3 (95% CI, 78.8-81.9) had excellent discrimination. The NAM model with AUROC of 76.1 (95% CI, 75.0-77.2) had acceptable discrimination. The area under the precision-recall curve demonstrated good performance exceeding the null of baseline prevalence (73.6 [95% CI, 72.3-74.8] for RAM, 23.1 [95% CI, 21.3-25.0] for NAM, and 39.5 [95% CI, 36.2-42.7] for YIM). The validation cohort had similar performance (eTable 6 in the Supplement).

**Figure.**
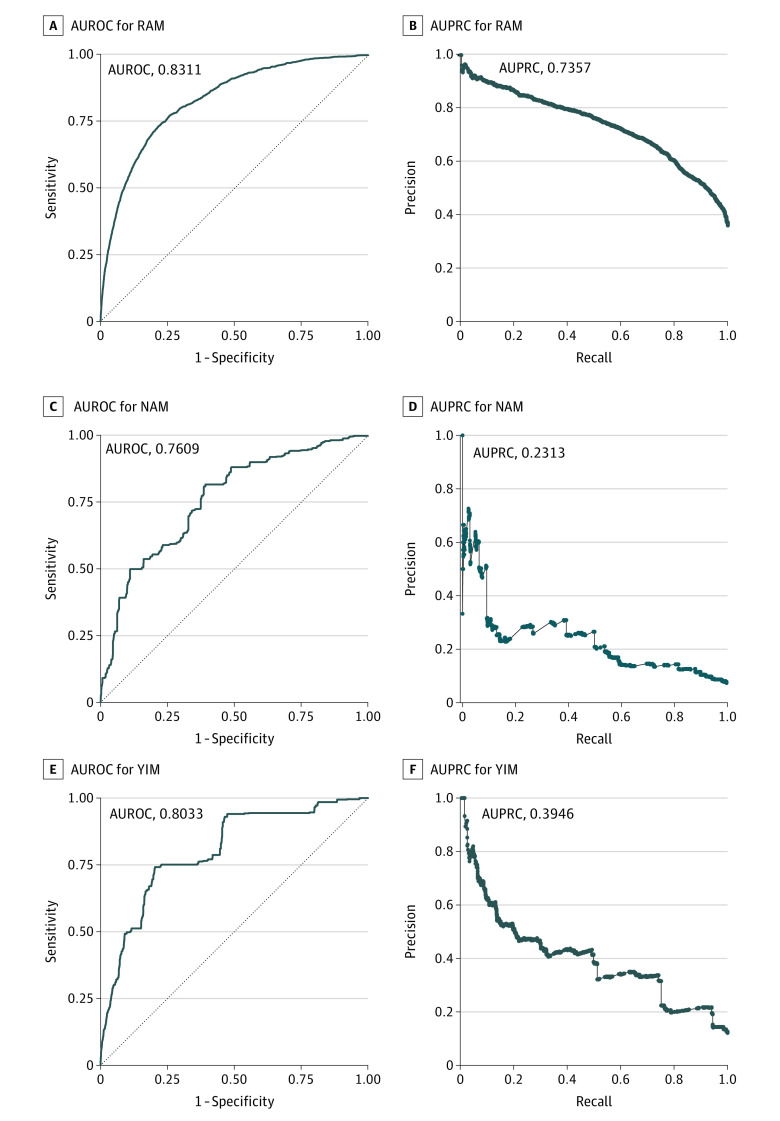
Area Under the Receiver Operating Characteristic Curve (AUROC) and Area Under the Precision-Recall Curve (AUPRC) for Recent Admission Model (RAM), New Admission Model (NAM), and Young Infant Model (YIM)

## Discussion

We derived and validated a suite of 3 complementary models of 30-day readmission risk for children, with good to excellent performance. By partitioning our cohort into 3 mutually exclusive subgroups, we accounted for heterogeneity of risk associated with age and prior utilization, overcoming limitations of previously published models.^15,16^ By distinguishing recent admissions from new admissions for children, we also revealed other predictive factors, particularly specific diagnoses, associated with readmission. Further, by developing and validating a readmission risk model for infants younger than 6 months, we provided the first known tool, to our knowledge, to estimate readmission risk separately for this age group. We incorporated indicators of severity of illness that can be applied to a hospitalization in real time (eg, prolonged LOS and procedures). Model variables are accessible in the EHR, permitting the potential for daily recalculation and offering the opportunity to mitigate readmission risk in real time.

Model discrimination was excellent overall, with *C* statistic values for both RAM and YIM that exceed many adult readmission risk prediction models.^8,9,10,11,12,13^ Our NAM cohort, which had the lowest *C* statistic of the models we developed, is still on par with another published pediatric model.^15,16^ In the validation data set, the *C* statistics remained strong for both the RAM and YIM cohorts, whereas they declined slightly in the NAM cohort but remained acceptable. Some of this decline may be related to smaller sample size of the validation set.

A significant strength of our models is that they were designed to be implemented in the EHR during a hospital stay and to change with clinical circumstances such as LOS and procedures, thereby enabling clinicians and case managers to assess risk of readmission when proactive measures can be taken. The previously available pediatric model^15^ relies on data that would not be available during hospitalization.

We hypothesized that risk factors for readmission would differ for children who had vs had not recently experienced hospitalization, which is why we developed and validated 2 separate models (RAM and NAM). This separation of models may explain why many of the SES factors we considered were not associated with readmission in final models, despite many prior studies demonstrating their importance as risk factors for pediatric readmission.^25,26^ Many SES factors we considered are likely stable during a 6-month period and may not be relevant for those with preceding care, as for the RAM cohort. In fact, only Black race and Hispanic ethnicity were associated with lower readmission risk in the cohort without prior utilization (NAM). Children in these groups may experience structural barriers to continuity of care, including greater fragmentation of health care, transportation barriers, and other sources of structural bias. Further research is warranted to elucidate factors that affect disparate access to postdischarge health care.

We also observed differences in the association between diagnosis and readmission risk between the RAM and NAM cohorts. For children with recent utilization, respiratory diagnoses were associated with lower odds of readmission. Other diagnoses, including sickle cell disease and CNS shunt infection or malfunction,^27,28^ which are well-documented risk factors for readmission, were not associated with readmission in this cohort. Again, this may be owing to the fact that these children are already in the RAM cohort with high risk for utilization (readmission rate >30%), and these diagnoses do not elevate readmission risk when compared with other children who also have high rates of reutilization. In contrast, within the NAM cohort, various diagnostic categories were associated with readmission. Chronic conditions, including sickle cell disease and CNS shunt, were associated with higher odds of readmission.

We expect that this suite of 3 models will help identify patients with postdischarge needs early in a hospital stay, promote better care coordination, and potentially overcome cognitive biases in identifying at-risk patients. Published postdischarge interventions for children include home nurse visits on all discharged patients^29^ and documentation of primary care clinician follow-up,^30^ which led to a counterintuitively higher reutilization rate in the intervention group, and postdischarge telephone calls,^31^ which had no effect. It is possible that such interventions were limited because they did not sufficiently differentiate levels of readmission risk. For instance, we found that although the RAM group represented 28.1% of encounters but 62.7% of readmissions, the NAM group represented 57.3% of encounters but only 26.3% of readmissions.

As such, we propose our models as a pragmatic and inclusive approach to triage case management resources based on objective estimates of readmission risk. Care management resources are currently allocated based on an underdeveloped, untested, highly variable framework. Readmission risk prediction would allow hospitals who implement a risk prediction model to allocate these scarce resources in a systematic way to children who are at increased risk of readmission. Application of these models may help shift the dialogue from the assessment of preventability of readmission toward the more proactive stance of discharge readiness. This systematic framework allows allocation of resources that may mitigate the health challenges that prompt readmission,^32^ acknowledging that readmission is a proxy for a broad range of difficulties pertaining to patients’ transitions across settings of care.

### Limitations

There are several limitations to this work. First, the model development and validation occurred at a single large children’s hospital, although the similarities in the covariates associated with readmission between the derivation and validation cohorts indicate a level of stability of our estimates. Our single-site approach also means we did not capture potential readmissions to other institutions. Second, our retrospective approach to model derivation may imperfectly apply during implementation in the EHR. Operationalization will require finding variable fields during the hospital stay in the active medical record, which may differ across institutions. Third, we did not exhaustively include all diagnoses, because we believed those with diminishing frequencies would have a lower likelihood of influencing model performance. A more automated method of including all diagnoses and aggregating them as proposed by Jovanovic et al^33^ may be integrated in future models. Fourth, the findings related to SES and race and ethnicity should be interpreted with caution, because they may be subject to inaccuracies and limitations in hospital-acquired data coding or other factors not fully understood at this time. Although neighborhood SES factors are commonly used to estimate individual SES risk^34,35^ and do correlate with individual SES factors in certain populations of children,^36^ neighborhood factors are a crude approximation for individual’s experiences and may contribute to the lack of retention of SES variables in our models; therefore they are a limitation.

## Conclusions

In this prognostic study, we developed and validated a suite of 3 complementary and inclusive pediatric readmission risk models, which had good to excellent discrimination and offer advantages compared with previously published models. All predictive factors in the models can be determined during pediatric hospitalization, allowing for real-time implementation in the future and the potential of discharge resource allocation toward higher-risk patients in real time.

## Notes

Supplement. **eMethods 1.** Treatment of Patients Who Died **eMethods 2.** Missing Data **eMethods 3.** Diagnoses **eTable 1.** Primary Diagnoses, Population 6 Months and Older **eTable 2.** Population Hospitalization Demographics **eTable 3.** Recent Admission Model (RAM), Bivariate **eTable 4.** New Admission Model (NAM), Bivariate **eTable 5.** Young Infant Model (YIM), Bivariate **eTable 6.** Model Performance by Area Under the Curve
