# Supplementary material for: Development and Validation of an Integrated Suite of Prediction Models for All-Cause 30-Day Readmissions of Children and Adolescents Aged 0 to 18 Years
Source: JAMA Netw Open. 2022 Nov 11;5(11):e2241513. doi: 10.1001/jamanetworkopen.2022.41513 (PMC9652755; doi:10.1001/jamanetworkopen.2022.41513)
Supplement: Supplement. — eMethods 1. Treatment of Patients Who Died eMethods 2. Missing Data eMethods 3. Diagnoses eTable 1. Primary Diagnoses, Population 6 Months and Older eTable 2. Population Hospitalization Demographics eTable 3. Recent Admission Model (RAM), Bivariate eTable 4. New Admission Model (NAM), Bivariate eTable 5. Young Infant Model (YIM), Bivariate eTable 6. Model Performance by Area Under the Curve [file jamanetwopen-e2241513-s001.pdf]

Supplementary Online Content

Goodman DM, Casale MT, Rychlik K, et al. Development and validation of an integrated suite of prediction models for all-cause 30-day readmissions of children and adolescents aged 0 to 18 years. *JAMA Netw Open*. 2022;5(11):e2241513. doi:10.1001/jamanetworkopen.2022.41513

eMethods 1. Treatment of Patients Who Died
eMethods 2. Missing Data
eMethods 3. Diagnoses
eTable 1. Primary Diagnoses, Population 6 Months and Older
eTable 2. Population Hospitalization Demographics
eTable 3. Recent Admission Model (RAM), Bivariate
eTable 4. New Admission Model (NAM), Bivariate
eTable 5. Young Infant Model (YIM), Bivariate
eTable 6. Model Performance by Area Under the Curve

This supplementary material has been provided by the authors to give readers additional information about their work.

© 2022 Goodman DM et al. *JAMA Network Open*.

eMethods 1. Treatment of Patients Who Died

We treated patients who died during admission differently in the derivation and validation datasets. In the derivation dataset, we included patients who died during an admission (259 deaths among 51,421 visits [0.5%]) in order to ascertain risk of readmission for the prior hospitalization, but we removed their final admission from subsequent risk prediction because risk would be zero. In the validation dataset, we retained patients who died during admission to simulate data as it would naturally populate the EHR.

eMethods 2. Missing Data

Data were missing in <5% of admissions. Missing data were categorized as 'other' for ethnicity/race, preferred language, insurance, principal diagnosis and surgical/medical admission. ZIP code-based variables (SES and distance to LCH) with missing values were assigned to the lower SES and furthest from LCH categories, respectively. No data were missing for sex or age; all other variables were derived from the EHR.

eMethods 3. Diagnoses

We followed a stepwise algorithm to identify the most relevant diagnostic categories:

- Step 1: For RAM and NAM, we selected the 20 most frequent diagnoses for subsequently readmitted patients.
- Step 2: We identified the 20 most frequent diagnoses for index hospitalizations in the whole cohort, reflecting the undifferentiated patient group for whom subsequent readmission risk is unknown.
- Step 3: Recognizing substantial diagnostic overlap, we identified the 11 most frequent diagnoses related to admission and readmission.
- Step 4: For YIM, and purposes of symmetry across models, we similarly grouped clinically relevant categories for admissions in the derivation data and identified the 11 most frequent diagnoses among all admissions.

© 2022 Goodman DM et al. *JAMA Network Open*.

eTable 1. Primary Diagnoses, Population 6 Months and Older

Diagnosis Group	ICD Code	Description	ICD Code Set
Appendicitis	K35.20	Acute appendicitis with generalized peritonitis	10
	K35.3	Acute appendicitis with localized peritonitis	10
	K35.33	Acute appendicitis with peritoneal abscess	10
	K35.80	Acute appendicitis without mention of peritonitis	10
	K35.80	Unspecified acute appendicitis	10
	K35.89	Other acute appendicitis	10
	K36	Other appendicitis	10
	K37	Appendicitis, unqualified	10
	K37	Unspecified appendicitis	10
	K38.1	Appendicular concretions	10
	K38.9	Other and unspecified diseases of appendix	10
Asthma	J44.9	Chronic obstructive pulmonary disease, unspecified	10
	J45.20	Mild intermittent asthma, uncomplicated	10
	J45.21	Mild intermittent asthma with (acute) exacerbation	10
	J45.22	Mild intermittent asthma with status asthmaticus	10
	J45.30	Mild persistent asthma, uncomplicated	10
	J45.31	Mild persistent asthma with (acute) exacerbation	10
	J45.32	Mild persistent asthma with status asthmaticus	10
	J45.40	Moderate persistent asthma, uncomplicated	10
	J45.41	Moderate persistent asthma with (acute) exacerbation	10
	J45.42	Moderate persistent asthma with status asthmaticus	10
	J45.51	Severe persistent asthma with (acute) exacerbation	10
	J45.52	Severe persistent asthma with status asthmaticus	10
	J45.901	Unspecified asthma with (acute) exacerbation	10
	J45.902	Unspecified asthma with status asthmaticus	10
	J45.909	Unspecified asthma, uncomplicated	10
	R06.2	Wheezing	10

© 2022 Goodman DM et al. *JAMA Network Open*.

Diagnosis Group	ICD Code	Description	ICD Code Set
CNS shunt	T82.598A	Other mechanical complication of other cardiac and vascular devices and implants, initial encounter	10
	T85.01XA	Breakdown (mechanical) of ventricular intracranial (communicating) shunt, initial encounter	10
	T85.09XA	Other mechanical complication of ventricular intracranial (communicating) shunt, initial encounter	10
	T85.615A	Breakdown (mechanical) of other nervous system device, implant or graft, initial encounter	10
	T85.695A	Mechanical complication of nervous system device, implant, and graft	10
	T85.738A	Infection and inflammatory reaction due to nervous system device, implant, and graft	10
	T85.890A	Other complications due to nervous system device, implant, and graft	10
	T85.890A	Other specified complication of nervous system prosthetic devices, implants and grafts, initial encounter	10
Dehydration / GI infection	A04.5	Intestinal infection due to campylobacter	10
	A04.5	Intestinal infection due to campylobacter	10
	A04.72	Intestinal infection due to Clostridium difficile	10
	A08.4	Viral intestinal infection, unspecified	10
	A08.8	Intestinal infection due to other organism, not elsewhere classified	10
	E86.0	Dehydration	10
	P74.1	Dehydration of newborn	10
	R19.7	Diarrhea	10
Fever	R50.9	Fever, unspecified	10
Respiratory, Lower (Bronchiolitis)	B97.4	Respiratory syncytial virus (RSV)	10
	B97.4	Respiratory syncytial virus as the cause of diseases classified elsewhere	10
	B97.89	Rhinovirus infection in conditions classified elsewhere and of unspecified site	10
	J12.1	Pneumonia due to respiratory syncytial virus	10
	J12.1	Respiratory syncytial virus pneumonia	10
	J12.3	Human metapneumovirus pneumonia	10
	J21.0	Acute bronchiolitis due to respiratory syncytial virus	10
	J21.0	Acute bronchiolitis due to respiratory syncytial virus (RSV)	10
	J21.1	Acute bronchiolitis due to human metapneumovirus	10
	J21.8	Acute bronchiolitis due to other infectious organisms	10
	J21.8	Acute bronchiolitis due to other specified organisms	10

© 2022 Goodman DM et al. *JAMA Network Open*.

**Diagnosis Group**	**ICD Code**	**Description**	**ICD Code Set**
	J21.9	Acute bronchiolitis, unspecified	10
Respiratory, Lower (Pneumonia)	A37.01	Whooping cough due to bordetella pertussis with pneumonia	10
	B39.2	Histoplasma capsulatum pneumonia	10
	J09.X1	Influenza due to identified novel influenza A virus with pneumonia	10
	J10.00	Influenza due to other identified influenza virus with unspecified type of pneumonia	10
	J10.08	Influenza due to other identified influenza virus with other specified pneumonia	10
	J10.1	Influenza due to other identified influenza virus with other respiratory manifestations	10
	J11.00	Influenza due to unidentified influenza virus with unspecified type of pneumonia	10
	J11.00	Influenza with pneumonia	10
	J11.08	Influenza due to unidentified influenza virus with specified pneumonia	10
	J11.1	Influenza due to unidentified influenza virus with other respiratory manifestations	10
	J11.89	Influenza with other manifestations	10
	J12.0	Adenoviral pneumonia	10
	J12.0	Pneumonia due to adenovirus	10
	J12.2	Parainfluenza virus pneumonia	10
	J12.2	Pneumonia due to parainfluenza virus	10
	J12.89	Other viral pneumonia	10
	J12.89	Pneumonia due to other virus not elsewhere classified	10
	J12.9	Viral pneumonia, unspecified	10
	J13	Pneumococcal pneumonia (streptococcus pneumoniae pneumonia)	10
	J13	Pneumonia due to Streptococcus pneumoniae	10
	J14	Pneumonia due to Hemophilus influenzae (H. influenzae)	10
	J15.0	Pneumonia due to Klebsiella pneumoniae	10
	J15.1	Pneumonia due to Pseudomonas	10
	J15.20	Pneumonia due to Staphylococcus, unspecified	10
	J15.211	Methicillin susceptible pneumonia due to Staphylococcus aureus	10
	J15.211	Pneumonia due to methicillin susceptible Staphylococcus aureus	10
	J15.212	Methicillin resistant pneumonia due to Staphylococcus aureus	10
	J15.212	Pneumonia due to methicillin resistant Staphylococcus aureus	10

© 2022 Goodman DM et al. *JAMA Network Open*.

Diagnosis Group	ICD Code	Description	ICD Code Set
	J15.4	Pneumonia due to other streptococci	10
Respiratory, Lower (Pneumonia), cont.	J15.4	Pneumonia due to streptococcus, group A	10
	J15.6	Pneumonia due to other Gram-negative bacteria	10
	J15.7	Pneumonia due to Mycoplasma pneumoniae	10
	J15.9	Bacterial pneumonia, unspecified	10
	J15.9	Unspecified bacterial pneumonia	10
	J18.0	Bronchopneumonia, unspecified organism	10
	J18.1	Lobar pneumonia, unspecified organism	10
	J18.8	Other pneumonia, unspecified organism	10
	J18.9	Pneumonia, organism unspecified(486)	10
	J18.9	Pneumonia, unspecified organism	10
	J69.0	Pneumonitis due to inhalation of food and vomit	10
	J69.0	Pneumonitis due to inhalation of food or vomitus	10
	J80	Acute respiratory distress syndrome	10
	J95.851	Ventilator associated pneumonia	10
	J96.00	Acute respiratory failure	10
	J96.00	Acute respiratory failure, unspecified whether with hypoxia or hypercapnia	10
	J96.01	Acute respiratory failure with hypoxia	10
	J96.20	Acute and chronic respiratory failure	10
	J96.21	Acute and chronic respiratory failure with hypoxia	10
	P23.9	Congenital pneumonia	10
	P23.9	Congenital pneumonia, unspecified	10
Respiratory, Upper	A37.90	Whooping cough, unspecified organism	10
	J05.0	Acute obstructive laryngitis (croup)	10
	J05.0	Croup	10
	J06.9	Acute upper respiratory infection, unspecified	10
	J06.9	Acute upper respiratory infections of unspecified site	10

© 2022 Goodman DM et al. *JAMA Network Open*.

Diagnosis Group	ICD Code	Description	ICD Code Set
Seizure	F44.5	Conversion disorder with seizures or convulsions	10
	G40.001	Localization-related (focal) (partial) idiopathic epilepsy and epileptic syndromes with seizures of localized onset, not intractable, with status epilepticus	10
	G40.009	Localization-related (focal) (partial) idiopathic epilepsy and epileptic syndromes with seizures of localized onset, not intractable, without status epilepticus	10
	G40.011	Localization-related (focal) (partial) idiopathic epilepsy and epileptic syndromes with seizures of localized onset, intractable, with status epilepticus	10
	G40.019	Localization-related (focal) (partial) idiopathic epilepsy and epileptic syndromes with seizures of localized onset, intractable, without status epilepticus	10
	G40.101	Localization-related (focal) (partial) symptomatic epilepsy and epileptic syndromes with simple partial seizures, not intractable, with status epilepticus	10
	G40.109	Epilepsia partialis continua without mention of intractable epilepsy	10
	G40.109	Localization-related (focal) (partial) epilepsy and epileptic syndromes with simple partial seizures, without mention of intractable epilepsy	10
	G40.109	Localization-related (focal) (partial) symptomatic epilepsy and epileptic syndromes with simple partial seizures, not intractable, without status epilepticus	10
	G40.111	Localization-related (focal) (partial) symptomatic epilepsy and epileptic syndromes with simple partial seizures, intractable, with status epilepticus	10
	G40.119	Epilepsia partialis continua with intractable epilepsy	10
	G40.119	Localization-related (focal) (partial) epilepsy and epileptic syndromes with simple partial seizures, with intractable epilepsy	10
	G40.119	Localization-related (focal) (partial) symptomatic epilepsy and epileptic syndromes with simple partial seizures, intractable, without status epilepticus	10
	G40.201	Localization-related (focal) (partial) symptomatic epilepsy and epileptic syndromes with complex partial seizures, not intractable, with status epilepticus	10
	G40.209	Localization-related (focal) (partial) epilepsy and epileptic syndromes with complex partial seizures, without mention of intractable epilepsy	10
	G40.209	Localization-related (focal) (partial) symptomatic epilepsy and epileptic syndromes with complex partial seizures, not intractable, without status epilepticus	10
	G40.211	Localization-related (focal) (partial) symptomatic epilepsy and epileptic syndromes with complex partial seizures, intractable, with status epilepticus	10

© 2022 Goodman DM et al. *JAMA Network Open*.

Diagnosis Group	ICD Code	Description	ICD Code Set
	G40.219	Localization-related (focal) (partial) epilepsy and epileptic syndromes with complex partial seizures, with intractable epilepsy	10
	G40.219	Localization-related (focal) (partial) symptomatic epilepsy and epileptic syndromes with complex partial seizures, intractable, without status epilepticus	10
	G40.301	Generalized idiopathic epilepsy and epileptic syndromes, not intractable, with status epilepticus	10
Seizure, cont.	G40.309	Generalized convulsive epilepsy without mention of intractable epilepsy	10
	G40.309	Generalized idiopathic epilepsy and epileptic syndromes, not intractable, without status epilepticus	10
	G40.309	Generalized nonconvulsive epilepsy without mention of intractable epilepsy	10
	G40.311	Generalized idiopathic epilepsy and epileptic syndromes, intractable, with status epilepticus	10
	G40.319	Generalized convulsive epilepsy with intractable epilepsy	10
	G40.319	Generalized idiopathic epilepsy and epileptic syndromes, intractable, without status epilepticus	10
	G40.319	Generalized nonconvulsive epilepsy with intractable epilepsy	10
	G40.401	Epileptic grand mal status	10
	G40.401	Other generalized epilepsy and epileptic syndromes, not intractable, with status epilepticus	10
	G40.409	Other generalized epilepsy and epileptic syndromes, not intractable, without status epilepticus	10
	G40.411	Other generalized epilepsy and epileptic syndromes, intractable, with status epilepticus	10
	G40.419	Other generalized epilepsy and epileptic syndromes, intractable, without status epilepticus	10
	G40.801	Other epilepsy, not intractable, with status epilepticus	10
	G40.802	Other epilepsy, not intractable, without status epilepticus	10
	G40.802	Other forms of epilepsy and recurrent seizures without mention of intractable epilepsy	10
	G40.803	Other epilepsy, intractable, with status epilepticus	10
	G40.804	Other epilepsy, intractable, without status epilepticus	10
	G40.804	Other forms of epilepsy and recurrent seizures with intractable epilepsy	10
	G40.811	Lennox-Gastaut syndrome, not intractable, with status epilepticus	10
	G40.813	Lennox-Gastaut syndrome, intractable, with status epilepticus	10
	G40.814	Lennox-Gastaut syndrome, intractable, without status epilepticus	10
	G40.821	Epileptic spasms, not intractable, with status epilepticus	10
	G40.822	Epileptic spasms, not intractable, without status epilepticus	10
	G40.822	Infantile spasms without mention of intractable epilepsy	10
	G40.823	Epileptic spasms, intractable, with status epilepticus	10

© 2022 Goodman DM et al. *JAMA Network Open*.

Diagnosis Group	ICD Code	Description	ICD Code Set
	G40.824	Epileptic spasms, intractable, without status epilepticus	10
	G40.824	Infantile spasms with intractable epilepsy	10
	G40.89	Other seizures	10
	G40.901	Epilepsy, unspecified, not intractable, with status epilepticus	10
Seizure, cont.	G40.909	Epilepsy, unspecified, not intractable, without status epilepticus	10
	G40.909	Unspecified epilepsy without mention of intractable epilepsy	10
	G40.911	Epilepsy, unspecified, intractable, with status epilepticus	10
	G40.919	Epilepsy, unspecified, intractable, without status epilepticus	10
	G40.919	Unspecified epilepsy with intractable epilepsy	10
	G40.A01	Epileptic petit mal status	10
	G40.A09	Absence epileptic syndrome, not intractable, without status epilepticus	10
	G40.B09	Juvenile myoclonic epilepsy, not intractable, without status epilepticus	10
	P90	Convulsions in newborn	10
	R56.00	Febrile convulsions (simple), unspecified	10
	R56.00	Simple febrile convulsions	10
	R56.01	Complex febrile convulsions	10
	R56.1	Post traumatic seizures	10
	R56.9	Other convulsions	10
	R56.9	Unspecified convulsions	10
Sickle cell	D56.4	Hereditary persistence of fetal hemoglobin (HPFH)	10
	D57.00	Hb-SS disease with crisis	10
	D57.00	Hb-SS disease with crisis, unspecified	10
	D57.01	Hb-SS disease with acute chest syndrome	10
	D57.02	Hb-Ss disease with splenic sequestration	10
	D57.03	Hb-ss disease with cerebral vascular involvement	10
	D57.09	Hb-ss disease with crisis with other specified complication	10
	D57.1	Hb-SS disease without crisis	10
	D57.1	Other sickle-cell disease without crisis	10
	D57.1	Sickle-cell disease without crisis	10

© 2022 Goodman DM et al. *JAMA Network Open*.

Diagnosis Group	ICD Code	Description	ICD Code Set
	D57.1	Sickle-cell disease, unspecified	10
	D57.20	Sickle-cell/Hb-C disease without crisis	10
	D57.211	Sickle-cell/Hb-C disease with acute chest syndrome	10
	D57.212	Sickle-cell/Hb-C disease with splenic sequestration	10
Sickle cell, cont.	D57.213	Sickle-cell/hb-c disease with cerebral vascular involvement	10
	D57.218	Sickle-cell/hb-c disease with crisis with other specified complication	10
	D57.219	Sickle-cell/Hb-C disease with crisis	10
	D57.219	Sickle-cell/Hb-C disease with crisis, unspecified	10
	D57.219	Sickle-cell/Hb-C disease with crisis, unspecified	10
	D57.413	Sickle-cell thalassemia, unspecified, with cerebral vascular involvement	10
	D57.418	Sickle-cell thalassemia, unspecified, with crisis with other specified complication	10
	D57.419	Sickle-cell thalassemia, unspecified, with crisis	10
	D57.431	Sickle-cell thalassemia beta zero with acute chest syndrome	10
	D57.432	Sickle-cell thalassemia beta zero with splenic sequestration	10
	D57.433	Sickle-cell thalassemia beta zero with cerebral vascular involvement	10
	D57.438	Sickle-cell thalassemia beta zero with crisis with other specified complication	10
	D57.439	Sickle-cell thalassemia beta zero with crisis, unspecified	10
	D57.451	Sickle-cell thalassemia beta plus with acute chest syndrome	10
	D57.452	Sickle-cell thalassemia beta plus with splenic sequestration	10
	D57.453	Sickle-cell thalassemia beta plus with cerebral vascular involvement	10
	D57.458	Sickle-cell thalassemia beta plus with crisis with other specified complication	10
	D57.459	Sickle-cell thalassemia beta plus with crisis, unspecified	10
	D57.811	Other sickle-cell disorders with acute chest syndrome	10
	D57.812	Other sickle-cell disorders with splenic sequestration	10
	D57.813	Other sickle-cell disorders with cerebral vascular involvement	10
	D57.818	Other sickle-cell disorders with crisis with other specified complication	10
	D57.819	Other sickle-cell disorders with crisis, unspecified	10
	D57.453	Sickle-cell thalassemia beta plus with cerebral vascular involvement	10

© 2022 Goodman DM et al. *JAMA Network Open*.

eTable 2. Population Hospitalization Demographics

		Derivation Cohort N=48,019	Validation Cohort N=17,486
		N (%)	N (%)
Primary Outcome	No subsequent admission within 30 days	40,242 (83.8)	14,731 (84.2)
	Subsequent admission within 30 days	7,777(16.2)	2,755 (15.8)
Age at Admission[1]	Less than 2 months	3,871 (8.1)	1,393 (8.0)
	2 to 5 months	3,127 (6.5)	1,089 (6.2)
	6 to 11 months	3,474 (7.2)	1,229 (7.0)
	1 to 4 years	13,589 (28.3)	5,139 (29.4)
	5 to 14 years	16,360 (34.1)	5,795 (33.1)
	15 years or older	7,598 (15.8)	2,841 (16.2)
Cardiac Critical Care Unit Admission	No	45,733 (95.2)	16,947 (96.9)
	Yes	1,973 (4.1)	539 (3.1)
Emergency Dept Admittance	No	21,698 (45.2)	7,824 (44.7)
	Yes	26,321 (54.8)	9,662 (55.3)
Insurance	Government[2]	25,975 (54.1)	9,418 (53.9)
	Commerical[3]	21,606 (45.0)	7,926 (45.3)
	Other or unspecified	438 (0.9)	142 (0.8)
Length of Stay	0 to 2 days	21,266 (61.6)	7,547 (59.7)
	0 to 3 days	8,027 (59.5)	2,740 (56.6)
	3 or more days	13,263 (38.4)	5,096 (40.3)
	4 or more days	5,463 (40.5)	2,103 (43.4)
PICU Admission, current	No	46,046 (95.9)	16,917 (56.9)
	Yes	1,973 (4.1)	539 (3.1)
Primary Care Physician Assigned	No	6,298 (13.1)	2,622 (15.0)
	Yes	41,721 (86.9)	14,864 (85.0)
(0.3)Primary Diagnosis Category	ALTE/BRUE	135 (0.3)	64 (0.4)
	Appendicitis	926 (1.9)	76 (0.4)
	Asthma	2,964 (6.2)	948 (5.4)
	Cardiac	406 (0.8)	156 (0.8)
	CNS shunt	344 (0.7)	115 (0.7)
	Congenital anomalies	472 (1.0)	186 (1.1)
	Dehydration/GI infection	1,524 (3.2)	527 (3.0)
	Esophageal reflux	132 (0.3)	37 (0.2)
	Fever	587 (1.2)	223 (1.3)
	Neonatal jaundice	240 (0.5)	84 (0.5)
	Pyloric stenosis	151 (0.3)	48 (0.3)
	Respiratory, Lower (Bronchiolitis or Pneumonia)	1,326 (2.8)	510 (2.9)
	Respiratory, Lower (Bronchiolitis)	1,840 (3.8)	486 (2.8)

© 2022 Goodman DM et al. *JAMA Network Open*.

		Derivation Cohort N=48,019	Validation Cohort N=17,486
		N (%)	N (%)
	Respiratory, Lower (Pneumonia)	2,437 (5.1)	1,503 (8.6)
	Respiratory, Upper	1,127 (2.3)	36 (2.1)
	Seizure	2,149 (4.5)	680 (3.9)
	Sickle cell	564 (1.2	230 (1.3
	UTI and pyelonephritis	196 (0.4)	60 (0.3)
	Other	30,499 (63.5)	11,189 (64.0)
Prior Procedure	No select procedures in the 6 months prior to admission	38,504 (80.2)	14,007 (80.1)
	One or more select procedures	9,515 (19.8)	3,479 (19.9)
Prior Utilization[4]	No utilization in the 6 months prior to admission	28,717 (59.8)	10,587(60.5)
	1	8,652 (18.0)	3,130 (17.9)
	1 or more		
	2	3,955 (8.2)	1,439 (8.2)
	3	2,157 (4.5)	744 (4.4)
	3 or more		
	4 or more	4,538 (9.5)	1,556 (8.9)
Race/Ethnicity	Black, non-Hispanic	8,318 (17.3)	3,000(17.2)
	Hispanic, Any Race	16,628 (34.6)	5,972 (34.2)
	White, non-Hispanic	18,360 (38.2)	6,625 (37.9)
	Other[6], non-Hispanic or unknown/unspecified	4,713 (9.8)	1,889 (10.8)
Season of Admission	Dec, Jan, Feb	12,682 (26.4)	4,482 (25.6)
	Mar, Apr, May	12,414 (25.9)	4,409 (25.2)
	Jun, Jul, Aug	11,109 (23.1)	3,979 (22.8)
	Sep, Oct, Nov	11,814 (24.6)	4,616 (26.4)
Socioeconomic Indicator[5], Education	Less than 90% with HS degree or higher, or unknown	27,562 (57.4)	10,028 (57.3)
	90%+ with HS degree or higher	20,247 (42.6)	7,458 (42.7)
Socioeconomic Indicator[5], Income	Less than $50,000 or unknown	12,907 (26.9)	4,639 (26.5)
	$50,000+	35,112 (73.1)	12,847 (73.5)
Surgical Medical Category	Medical	37,297 (77.7)	14,346 (82.0)
	Surgical	10,722 (22.3)	3,140 (18.0)

[1] Age was aggregated into clinically relevant categories in RAM and NAM. For YIM, the age cut point was established empirically based on age distribution during initial bivariate analysis
[2] Includes Blue Cross, Managed Care or other commercial insurance
[3] Includes Medicare, Medicaid, ACE, CCE, Champus or other government insurance
[4] Utilization is defined as an inpatient stay, observation, or ED visit. In the New Admission Model, prior utilization pertains to ED visits only, since by definition this population includes only encounters for which there is no previous hospitalization
[5] Based on zip code
[6] Other race includes: American Indian/Alaskan Native; Asian; Declined; Native Hawaiian/Pacific Islander; Other; Unknown

© 2022 Goodman DM et al. *JAMA Network Open*.

eTable 3. Recent Admission Model (RAM), Bivariate

RAM Independent Variables		Estimate (SE)	OR (95% CI)	P-Value
Age at Admission[1]	6 to 11 months	0.10 (0.09)	1.10 (0.92, 1.33)	0.28
	1 to 4 years	-0.09 (0.07)	0.91 (0.80, 1.04)	0.16
	15 years or older	0.17 (0.08)	1.19 (1.03, 1.38)	0.02
	5 to 14 years (reference)	0		
Cardiac Critical Care Unit Admission	Yes	-0.12 (0.13)	0.89 (0.70, 1.14)	0.35
	No (reference)	0		
Emergency Dept Admittance	No	0.22 (0.04)	1.25 (1.14, 1.36)	<0.001
	Yes (reference)	0		
Insurance	Commercial[2]	0.22 (0.06)	1.25 (1.12, 1.40)	<0.001
	Other or unspecified	-0.30 (0.29)	0.74 (0.42, 1.31)	0.30
	Government[3] (reference)	0		
Length of Stay	4 or more days	0.26 (0.04)	1.30 (1.19, 1.41)	<0.001
	0 to 3 days (reference)	0		
PICU Admission, current	Yes	-0.14 (0.11)	0.87 (0.71, 1.07)	0.19
	No (reference)	0		
Primary Care Physician Assigned	No	0.15 (0.11)	1.16 (0.95, 1.43)	0.15
	Yes (reference)	0		
Primary Diagnosis Category	Appendicitis	-0.88 (0.37)	0.41 (0.20,0.85)	0.02
	Asthma	-1.46 (0.16)	0.23 (0.17,0.32)	<0.001
	CNS shunt	-0.30 (0.20)	0.74 (0.50,1.10)	0.13
	Dehydration/GI infection	-0.28 (0.13)	0.76 (0.59,0.97)	0.03
	Fever	-0.25 (0.18)	0.78 (0.55,1.11)	0.17
	Respiratory, Lower (Bronchiolitis)	-0.60 (0.13)	0.55 (0.42,0.71)	<0.001
	Respiratory, Lower (Pneumonia)	-0.37 (0.10)	0.69 (0.58,0.84)	<0.001
	Respiratory, Upper	-0.20 (0.16)	0.82 (0.59,1.13)	0.22
	Seizure	-0.74 (0.12)	0.48 (0.38,0.60)	<0.001
	Sickle cell	-0.32 (0.19)	0.73 (0.50,1.05)	0.09
	Other (reference)	0		

© 2022 Goodman DM et al. *JAMA Network Open*.

RAM Independent Variables		Estimate (SE)	OR (95% CI)	P-Value
Prior Procedure	One or more select procedures in the 6 months prior to admission	1.17 (0.05)	3.22 (2.93,3.546)	<0.001
	No select procedures (reference)	0		
Prior Utilization[4]	4 or more utilizations in the 6 months prior to admission	1.02 (0.06)	2.77 (2.47,3.10)	<0.001
	3	0.63 (0.07)	1.87 (1.64,2.14)	<0.001
	2	0.34 (0.06)	1.40 (1.25,1.58)	<0.001
	1 (reference)	0		
Race/Ethnicity	Black, non-Hispanic	-0.28 (0.08)	0.76 (0.64,0.89)	<0.001
	Hispanic, Any Race	-0.20 (0.07)	0.82 (0.72,0.94)	0.004
	White, non-Hispanic (reference)	0		
	Other[6], non-Hispanic or unknown/ unspecified	-0.02 (0.10)	0.98 (0.80,1.20)	0.82
Season of Admission	Dec, Jan, Feb	0.07 (0.06)	1.07 (0.95,1.20)	0.25
	Jun, Jul, Aug	0.02 (0.06)	1.03 (0.91,1.15)	0.68
	Sep, Oct, Nov	0.04 (0.06)	1.04 (0.92,1.17)	0.53
	Mar, Apr, May (reference)	0		
Socioeconomic Indicator[5], Education	90%+ with High School degree or higher	0.10 (0.06)	1.10 (0.99,1.23)	0.09
	Less than 90% with HS degree or higher, or unknown (reference)	0		
Socioeconomic Indicator[5], Income	Less than $50,000 or unknown	-0.16 (0.07)	0.86 (0.75,0.97)	0.02
	$50,000+ (reference)	0		
Surgical Medical Category	Surgical	-0.61 (0.07)	0.55 (0.48,0.63)	<0.001
	Medical (reference)	0		

[1] Age was aggregated into clinically relevant categories in RAM and NAM
[2] Includes Blue Cross, Managed Care or other commercial insurance
[3] Includes Medicare, Medicaid, ACE, CCE, Champus or other government insurance
[4] Utilization is defined as an inpatient stay, observation, or ED visit. In the New Admission Model, prior utilization pertains to ED visits only, since by definition this population includes only encounters for which there is no previous hospitalization
[5] Based on zip code
[6] Other race includes: American Indian/Alaskan Native; Asian; Declined; Native Hawaiian/Pacific Islander; Other; Unknown

© 2022 Goodman DM et al. *JAMA Network Open*.

eTable 4. New Admission Model (NAM), Bivariate

NAM Independent Variables		Estimate (SE)	OR (95% CI)	P-Value
Age at Admission[1]	6 to 11 months	0.16 (0.08)	1.17 (1.00,1.38)	0.06
	1 to 4 years	-0.12 (0.06)	0.89 (0.79,0.99)	0.03
	15 years or older	0.27 (0.06)	1.31 (1.15,1.48)	<0.001
	5 to 14 years (reference)	0		
Cardiac Critical Care Unit Admission	Yes	0.34 (0.11)	1.41 (1.15,1.74)	<0.001
	No (reference)	0		
Emergency Dept Admittance	No	0.10 (0.05)	1.11 (1.01,1.22)	0.03
	Yes (reference)	0		
Insurance	Commercial[2]	0.15 (0.05)	1.17 (1.07,1.28)	<0.001
	Other or unspecified	-0.22 (0.26)	0.80 (0.50,1.33)	0.40
	Government[3] (reference)	0		
Length of Stay	3 or more days	0.94 (0.05)	2.55 (2.33, 2.80)	<0.001
	0 to 2 days (reference)	0		
PICU Admission, current	Yes	0.30 (0.11)	1.34 (1.08,1.67)	0.008
	No (reference)	0		
Primary Care Physician Assigned	No	-0.21 (0.07)	0.81 (0.70, 0.93)	0.003
	Yes (reference)	0		
Primary Diagnosis Category	Appendicitis	-0.75 (0.17)	0.47 (0.34,0.66)	<0.001
	Asthma	-0.98 (0.12)	0.37 (0.30,0.47)	<0.001
	CNS shunt	0.62 (0.23)	1.87 (1.20,2.92)	0.006
	Dehydration/GI infection	-0.67 (0.15)	0.51 (0.38,0.68)	<0.001
	Fever	-0.05 (0.33)	0.95 (0.49,1.83)	0.88
	Respiratory, Lower (Bronchiolitis)	-0.36 (0.12)	0.70 (0.55,0.88)	0.002
	Respiratory, Lower (Pneumonia)	-0.20 (0.10)	0.82 (0.67,0.997)	0.05
	Respiratory, Upper	-0.57 (0.18)	0.57 (0.40,0.82)	0.002
	Seizure	-0.51 (0.12)	0.60 (0.48,0.76)	<0.001
	Sickle cell	0.57 (0.20)	1.77 (1.21,2.60)	0.004
	Other (reference)	0		

© 2022 Goodman DM et al. *JAMA Network Open*.

NAM Independent Variables		Estimate (SE)	OR (95% CI)	P-Value
Prior Procedure	One or more select procedures in the 6 months prior to admission	1.05 (0.07)	2.87 (2.51,3.28)	<0.001
	No select procedures (reference)	0		
Prior Utilization[4]	1 or more utilizations in the 6 months prior to admission	0.17 (0.06)	1.19 (1.05,1.34)	0.005
	0 (reference)	0		
Race/Ethnicity	Black, non-Hispanic	-0.24 (0.07)	0.79 (0.69,0.90)	<0.001
	Hispanic, Any Race	-0.25 (0.05)	0.78 (0.70,0.86)	<0.001
	White, non-Hispanic (reference)	0		
	Other[6], non-Hispanic or unknown/unspecified	-0.27 (0.09)	0.76 (0.65,0.90)	0.002
Season of Admission	Dec, Jan, Feb	0.04 (0.06)	1.04 (0.92,1.18)	0.53
	Jun, Jul, Aug	-0.09 (0.07)	0.92 (0.80,1.05)	0.21
	Mar, Apr, May	0.03 (0.06)	1.03 (0.91,1.17)	0.66
	Sep, Oct, Nov (reference)	0		
Socioeconomic Indicator[5], Education	90%+ with High School degree or higher	0.20 (0.05)	1.23 (1.12,1.34)	<0.001
	Less than 90% with HS degree or higher, or unknown (reference)	0		
Socioeconomic Indicator[5], Income	Less than $50,000 or unknown	-0.21 (0.05)	0.81 (0.73,0.90)	<0.001
	$50,000+ (reference)	0		
Surgical Medical Category	Surgical	-0.03 (0.05)	0.97 (0.87,1.07)	0.52
	Medical (reference)	0		

[1] Age was aggregated into clinically relevant categories in RAM and NAM
[2] Includes Blue Cross, Managed Care or other commercial insurance
[3] Includes Medicare, Medicaid, ACE, CCE, Champus or other government insurance
[4] Utilization is defined as an inpatient stay, observation, or ED visit. In the New Admission Model, prior utilization pertains to ED visits only, since by definition this population includes only encounters for which there is no previous hospitalization
[5] Based on zip code
[6] Other race includes: American Indian/Alaskan Native; Asian; Declined; Native Hawaiian/Pacific Islander; Other; Unknown

© 2022 Goodman DM et al. *JAMA Network Open*.

eTable 5. Young Infant Model (YIM), Bivariate

YIM Independent Variables		Estimate (SE)	OR (95% CI)	P-Value
Age at Admission[1]	2 to 5 months	0.38 (0.08)	1.46 (1.25,1.71)	<0.001
	less than 2 months (reference)	0		
Cardiac Critical Care Unit Admission	Yes	0.50 (0.12)	1.65 (1.30,2.09)	<0.001
	No (reference)	0		
Emergency Dept Admittance	No	0.32 (0.08)	1.38 (1.19,1.61)	<0.001
	Yes (reference)	0		
Insurance	Commercial[2]	-0.04 (0.08)	0.95 (0.81,1.12)	0.55
	Other or unspecified	-0.85 (0.77)	0.43 (0.10,1.92)	0.27
	Government[3] (reference)	0		
Length of Stay	3 or more days	0.65 (0.08)	1.92 (1.64,2.25)	<0.001
	0 to 2 days (reference)	0		
PICU Admission, current	Yes	0.67 (0.24)	1.95 (1.21,3.12)	0.006
	No (reference)	0		
Primary Care Physician Assigned	No	-0.03 (0.09)	0.97 (0.81,1.16)	0.75
	Yes (reference)	0		
Primary Diagnosis Category	ALTE/BRUE	-0.31 (0.31)	0.73 (0.40,1.34)	0.31
	Cardiac	0.35 (0.15)	1.42 (1.06,1.90)	0.02
	Congenital anomalies	-0.12 (0.16)	0.89 (0.65,1.21)	0.45
	Esophageal reflux	0.01 (0.27)	1.01 (0.60,1.72)	0.96
	Fever	-1.09 (0.29)	0.34 (0.19,0.59)	<0.001
	Neonatal jaundice	-1.56 (0.39)	0.21 (0.10,0.45)	<0.001
	Pyloric stenosis	-0.85 (0.36)	0.43 (0.21,0.86)	0.02
	Respiratory, Lower (Bronchiolitis and Pneumonia)	-0.46 (0.11)	0.63 (0.51,0.79)	<0.001
	Respiratory, Upper	-0.69 (0.25)	0.50 (0.30,0.83)	0.007
	UTI and pyelonephritis	-0.73 (0.29)	0.48 (0.27,0.86)	0.01
	Other (reference)	0		
Prior Procedure	One or more select procedures in the 6 months prior to admission	0.77 (0.09)	2.17 (1.81,2.61)	<0.001
	No select procedures (reference)	0		
Prior Utilization[4]	3 or more utilizations in the 6 months prior to admission	1.24 (0.15)	3.47 (2.57,4.69)	<0.001

© 2022 Goodman DM et al. *JAMA Network Open*.

YIM Independent Variables		Estimate (SE)	OR (95% CI)	P-Value
	2	0.95 (0.14)	2.60 (1.98,3.41)	<0.001
	1	0.47 (0.10)	1.61 (1.33,1.95)	<0.001
	0 (reference)	0		
Race/Ethnicity	Black, non-Hispanic	0.04 (0.12)	1.05 (0.83,1.32)	0.71
	Hispanic, Any Race	-0.03 (0.10)	0.97 (0.80,1.17)	0.72
	White, non-Hispanic (reference)	0		
	Other[6], non-Hispanic or unknown/unspecified	-0.06 (0.14)	0.95 (0.72,1.24)	0.69
Season of Admission	Jun, Jul, Aug	-0.003 (0.112)	1.00 (0.80,1.24)	0.98
	Mar, Apr, May	-0.09 (0.11)	0.91 (0.74,1.13)	0.41
	Sep, Oct, Nov	0.19 (0.10)	1.21 (0.99,1.49)	0.07
	Dec, Jan, Feb (reference)	0		
Socioeconomic Indicator[5], Education	90%+ with High School degree or higher	0.20 (0.08)	1.22 (1.04,1.43)	0.02
	Less than 90% with HS degree or higher, or unknown (reference)	0		
Socioeconomic Indicator[5], Income	Less than $50,000 or unknown	-0.06 (0.09)	0.94 (0.79,1.13)	0.52
	$50,000+ (reference)	0		
Surgical Medical Category	Surgical	-0.15 (0.11)	0.86 (0.69,1.06)	0.16
	Medical (reference)	0		

[1] Age cut point was established empirically based on age distribution in YIM
[2] Includes Blue Cross, Managed Care or other commercial insurance
[3] Includes Medicare, Medicaid, ACE, CCE, Champus or other government insurance
[4] Utilization is defined as an inpatient stay, observation, or ED visit. In the New Admission Model, prior utilization pertains to ED visits only, since by definition this population includes only encounters for which there is no previous hospitalization
[5] Based on zip code
[6] Other race includes: American Indian/Alaskan Native; Asian; Declined; Native Hawaiian/Pacific Islander; Other; Unknown

© 2022 Goodman DM et al. *JAMA Network Open*.

eTable 6. Model Performance by Area Under the Curve

	**Derivation Cohort (2016-2018)**	**Validation Cohort (2019)**
RAM	0.8311 (0.8241, 0.8382)	0.8208 (0.8086, 0.8329)
NAM	0.7609 (0.7503, 0.7715)	0.6848 (0.6655, 0.7042)
YIM	0.8033 (0.7877, 0.8189)	0.7558 (0.7255, 0.7860)

© 2022 Goodman DM et al. *JAMA Network Open*.
